# Lower cardiorespiratory fitness is associated with an altered gut microbiome. The Study of Health in Pomerania (SHIP)

**DOI:** 10.1038/s41598-025-88415-4

**Published:** 2025-02-12

**Authors:** Marcello Ricardo Paulista Markus, Frank-Ulrich Weiss, Johannes Hertel, Stefan Weiss, Malte Rühlemann, Corinna Bang, Andre Franke, Uwe Völker, Georg Homuth, Thomas Kocher, Henry Völzke, Markus M. Lerch, Till Ittermann, Stephan Burkhard Felix, Ralf Ewert, Martin Bahls, Marcus Dörr, Fabian Frost

**Affiliations:** 1 Department of Internal Medicine B, Cardiology, Angiology, Pneumology and Internal Intensive Care Medicine, University Medicine Greifswald, Ferdinand-Sauerbruch-Straße, 17475 Greifswald, Germany; 2 German Centre for Cardiovascular Research (DZHK), Partner site Greifswald, Greifswald, Germany; 3 German Center for Diabetes Research (DZD), Partner site Greifswald, Greifswald, Germany; 4 Department of Medicine A, University Medicine Greifswald, Greifswald, Germany; 5 Department of Psychiatry and Psychotherapy, University Medicine Greifswald, Greifswald, Germany; 6 Institute of Clinical Molecular Biology, Christian-Albrechts University of Kiel, Kiel, Germany; 7 Institute for Medical Microbiology and Hospital Epidemiology, Hannover Medical School, Hannover, Germany; 8 Department of Functional Genomics, Interfaculty Institute of Genetics and Functional Genomics, University Medicine Greifswald, Greifswald, Germany; 9 Unit of Periodontology, Department of Restorative Dentistry, Periodontology, Endodontology, and Preventive and Pediatric Dentistry, University Medicine Greifswald, Greifswald, Germany; 10 Department of Study of Health in Pomerania/Clinical-Epidemiological Research, Institute for Community Medicine, University Medicine Greifswald, Greifswald, Germany

**Keywords:** Cardiorespiratory fitness, Gut microbiome, Microbiota, Pathogen, Sedentarism, Cardiology, Gastroenterology

## Abstract

Sedentarism is characterized by low levels of physical activity, a risk factor for obesity and cardio-metabolic diseases. It can also adversely affect the composition and diversity of the gut microbiome which may result in harmful consequences for human health. While cardiorespiratory fitness (CRF) is inversely and independently associated with cardiovascular risk factors and diseases and all-cause mortality, the relationship between low CRF and the gut microbiome is not well known. A total of 3,616 individuals from two independent population-based cohorts of the Study of Health in Pomerania (SHIP-START and SHIP-TREND) performed standardized, symptom-limited cardiopulmonary exercise testing (CPET) and had faecal samples collected to determine gut microbiota profiles (16S rRNA gene sequencing). We analysed cross-sectional associations of CRF with the gut microbiome composition controlling for confounding factors. Lower CRF was associated with reduced microbial diversity, loss of beneficial short-chain fatty acid producing bacteria (i.e. *Butyricoccus*, *Coprococcus*, unclassified *Ruminococcaceae* or *Lachnospiraceae*) and an increase in opportunistic pathogens such as *Escherichia/Shigella*, or *Citrobacter*. Decreased cardiorespiratory performance was associated with a gut microbiota pattern that has been previously related to a proinflammatory state. These associations were independent of body weight or glycemic control.

**Supplementary Information:**

The online version contains supplementary material available at 10.1038/s41598-025-88415-4.

## Introduction

The intestinal microbiome comprises all the microorganisms that reside in our gut. Its composition depends on a variety of different host factors such as age, sex, genetics, diet, or body weight^1–3^. In addition, common disorders like obesity or diabetes mellitus have been shown to be associated with changes in the gut microbiome^4^. Conversely, the pathogenesis and progression of these metabolic disorders are also, in return, affected by the gut microbiota and their metabolic activity^5,6^.

A hypercaloric diet and sedentarism are the main drivers of the current pandemic of obesity and associated metabolic disorders. Therapeutic approaches include not only promotion of a balanced diet but also an increase in physical activity. Previous studies^7,8^, mostly conducted in healthy adults or athletes, showed that increased exercise or physical activity has an impact on the gut microbiome improving its composition, diversity and inflammatory potential. However, in the general population, a very different lifestyle-related constellation is predominant and largely characterized by a lack of regular exercise and low levels of physical activity^9^.

Noteworthy, sedentarism, and its subsequent pathophysiological mechanisms, are not necessarily the opposite of those related with physical activity or exercise training^10^. Cardiorespiratory fitness (CRF) is the ability of the cardiovascular and respiratory systems to deliver blood and oxygen as required by the body muscles during physical activity. Together with a genetic predisposition, regular physical activity and exercise training are the most important contributors for a good CRF^11^ which can be objectively quantified during cardiopulmonary exercise testing (CPET)^12^. CRF is inversely and independently associated with cardiovascular risk factors and diseases and all-cause mortality. Previously, we showed that lower CRF and muscular fitness may have direct detrimental effects on the heart^13–15^ and on the liver^16^. However, data on the impact of low CRF on the microbiome in large population-based cohorts is not available so far. To expand this knowledge, we have analysed differences in the gut microbiome associated with low CRF in two cohorts with a total of 3,616 participants of the population-based Study of Health in Pomerania (SHIP).

## Methods

### Study participants

The analysed data originate from the population-based Study of Health in Pomerania (SHIP)^17–19^. The study design and recruitment strategy have been described in detail elsewhere^17–19^. In brief, a random cluster sample (age range 20 to 79 years) was drawn from the population of West Pomerania, the north-eastern region of Germany. The net sample (without migrated or deceased persons) comprised 6,265 eligible individuals with 4,308 (2,193 women) of them participating in the baseline (SHIP-START-0) study (response 68.8%). All subjects who participated in the baseline SHIP were re-invited to take part in the first examination follow-up (SHIP-START-1), which was realized from 2002 to 2006. Of the 3,949 persons eligible for SHIP-START-1, 3,300 subjects were re-examined, resulting in a follow-up response of 83.6%. For the second examination follow-up (SHIP-START-2)^18,19^ conducted from 2008 to 2012, all 3,708 eligible individuals that participated in the baseline study were re-invited. Of them, 2,333 were re-examined (follow-up response of 62.9%).

While SHIP-START-2 was being conducted, between 2008 and 2012, a second independent cohort was established, called SHIP-TREND-0^18,19^, covering the same region as the initial SHIP. A stratified random sample of 8,826 adults, aged 20 to 79 years, was selected. Participation in the initial SHIP cohort was an exclusion criterion. In total, 4,420 individuals participated in SHIP-TREND-0 (response 50.1%). For the present study, we performed cross-sectional analyses using pooled data from SHIP-START-2 and SHIP-TREND-0 (*n* = 6,753 individuals; 3,510 women [52.0%]). Of these, 3,616 subjects (50.0% women) aged 20 to 90 years, performed cardiopulmonary exercise testing (CPET) and collected faecal samples for determination of gut microbiota. We excluded nine participants because of missing data regarding body mass index (BMI), glycated haemoglobin (HbA1c) measurements, smoking history or food frequency score (FFS). Further 49 subjects were excluded due to antibiotics intake at the time of sample collection. The final analytical sample comprised 3,558 participants (49.9% women; aged 20 to 90 years).

### Cardiopulmonary exercise testing

Using a calibrated electromagnetically braked cycle ergometer (Ergoselect 100, Ergoline, Germany) a symptom-limited exercise test was performed according to a modified Jones protocol (more exactly described below)^20^. All tests, accompanied by a physician, were performed at room air according to current guidelines for exercise testing, with continuous monitoring of electrocardiogram (twelve lead ECG was recorded during rest and every minute thereafter), blood pressure and oxygen saturation. Before the beginning of the test, the participants were encouraged to reach maximal exhaustion, but no further motivation was used during the exercise. The protocol included 3 min of rest followed by 1 min of unloaded cycling (20 Watts) at ~ 60 rpm. Thereafter, the workload was increased by 16 W every minute. In the absence of chest pain and/or electrocardiographic abnormalities, all tests were continued as symptom-limited (volitional exertion, dyspnea or fatigue) followed by 5 min of recovery. Exercise duration was investigated from the start of exercise (without the resting period) up to its termination.

### Gas exchange variables

Minute ventilation (V_E_), tidal volume, oxygen uptake (VO_2_) and carbon dioxide output (VCO_2_) and ventilatory variables were analysed breath-by-breath averaged over 10s intervals using the Oxycon Pro system (Jaeger/Viasys Healthcare, Hoechberg, Germany), together with a Rudolph’s mask, which was recalibrated before each test. Peak oxygen uptake (VO_2peak_) in L/min was defined as the highest 10s average of absolute VO_2_ during late exercise or early recovery. It is a strong predictor of mortality commonly used in the evaluation of patients for cardiac transplantation. Peak oxygen pulse (peak O_2_ pulse) in mL/beat was calculated as VO_2peak_ divided by peak heart rate (averaged during the highest 10 s average of absolute VO_2_ during late exercise or early recovery). It is an indicator of stroke volume and arteriovenous O_2_ difference used for predicting prognosis, including major cardiac events, in patients with systolic heart failure. Maximum working capacity (Watt_max_) in W was determined as the highest workload achieved during the CPET. It is used as an index of current status of physical training and progress in a subsequent training program. Oxygen uptake at the anaerobic threshold (VO_2_@AT) in mL/min was determined by revising the gas exchange analysis by assessing the relation of VO_2_ to VCO_2_ (V-slope method). The anaerobic threshold (AT) is the peak oxygen consumption where the energy demands surpass the circulatory ability to sustain an aerobic metabolism. Ventilatory efficiency (VE/VCO_2_ slope) was expressed by the minute ventilation changes as a function of the pulmonary VCO_2_. The ventilatory efficiency (minute ventilation required to eliminate carbon dioxide) during exercise is a strong predictor of major adverse cardiovascular events in heart failure patients and in the general population^20^.

### 16S rRNA gene sequencing

For determination of gut microbiota profiles, 16S rRNA gene sequencing of faecal samples was performed as described before in detail^1^. In brief, stool samples were collected in a tube containing DNA stabilizing EDTA buffer at home and transported to the study centre by mail or the study participants individually. The PSP Spin Stool DNA Kit (Stratec Biomedical AG, Birkenfeld, Germany) was used for isolation of DNA. All DNA isolates were stored at −20 °C until amplification of the V1 and V2 regions of bacterial 16S rRNA genes was performed using the primer pair 27 F and 338R. Sequencing was subsequently performed on a MiSeq platform (Illumina, San Diego, USA) using a dual-indexing approach.

### Assignment of taxonomy

MiSeq FastQ files were created with CASAVA 1.8.2 (https://support.illumina.com/sequencing/sequencing_software/casava). For amplicon-data processing the open-source software package DADA2 (v.1.10)^21^ was used following the authors’ recommended procedure for large datasets (https://benjjneb.github.io/dada2/bigdata.html), adapted to the targeted V1-V2 amplicon as described previously^22^. Briefly, on both reads, five bases were truncated from the 5′ end of the sequence to a length of 200 and 150 bp, respectively. A shorter resulting read length after truncation was possible if the sequence quality dropped below five. Read-pairs were discarded if they contained ambiguous bases, expected errors higher than two and when originating from PhiX spike-in. Error profiles were inferred using 1 million reads of the respective sequencing run, followed by dereplication, error correction and merging of forward and reverse reads. ASV abundance of tables of all samples were combined and the removeBimeraDenovo function was used (consensus mode) to identify and remove chimeric amplicon sequences. For taxonomic annotation a Bayesian classifier and the Ribosomal Database Project (RDP) training set version 16 were used. All data were rarefied to 10,000 reads per sample before computation of alpha or beta diversity or any between genus level analyses. Alpha diversity scores estimate the microbial variation within a given sample with higher scores indicating larger microbial diversity. Beta diversity scores assess the microbial variation or similarity between two samples, e.g. how different or similar they are.

### Phenotypic data

For the calculation of the BMI, the participants body weight in kilogram was divided by the square of the body height. To assess nutritional habits, data from fifteen food categories (meat, sausage, fish, boiled potatoes, pasta, rice, raw vegetables, boiled vegetables, fruits, whole grain or black or crisp bread, oats and cornflakes, eggs, cake or cookies, sweets, and savoury snacks) were used to calculate a food frequency score (FFS) as described elsewhere^23^. In brief, the frequency of food intake for each nutritional group was categorized as follows: ‘1’ = daily or almost daily, ‘2’ = several times per week, ‘3’ = once a week, ‘4’ = several times per month, ‘5’ = once a month or less, ‘6’ = never or almost never. The dietary patterns of each food group were then classified according to the recommendations of the German Society of Nutrition to 0 (unfavourable), 1 (normal) or 2 (recommended) points and summed up^24^. The FFS has a theoretical range from 0 to 30 while a higher score reflects a higher quality of diet.

### Data and statistical analysis

All statistical analyses were done in the software environment ‘R’ (v.3.3.3, https://www.R-project.org/). For creation of plots the packages ‘ggplot2’ and ‘ggraph’ were used. To estimate the contribution of each variable to the overall beta diversity permutational analyses of variance (function ‘adonis’, ‘vegan’ package) were performed based on a Bray-Curtis dissimilarity matrix (using a genus level abundance table) or weighted UniFrac distance (using an ASV level abundance table) and its statistical significance assessed with 1,000 permutations. For computation of the alpha diversity score ‘inverse Simpson’s index’ (function ‘diversity’, index = “invsimpson”, ‘vegan’ package) was performed using the function ‘diversity’ (‘vegan’ package) based on ASV abundance tables. Possible associations between CPET variables and individual taxa were investigated for all genera or families which were present in at least 10% of all samples using two different models. The first model was a negative binomial regression (function ‘glm.nb’, ‘MASS’ package) approach with the respective zero-truncated taxon as outcome and the respective cardiopulmonary function parameter as exposure. Outliers +/− five standard deviations were removed from each taxon before analysis. Only taxa with a presence of at least 10% of all samples were analysed. The second model was a logistic regression model (function ‘glm’, family = binomial, ‘stats’ package) with each of the respective CPET parameter as exposure and the respective binary (0 = absent vs. 1 = present) coded taxon as outcome. Only taxa with a presence of at least ten but not more than 80% were analysed in this model. Finally, we used a linear regression model (function ‘lm’, ‘stats’ package) to investigate possible associations between CPET parameters as exposure and the alpha diversity scores species richness (N0) or inverse Simpson’s index (N2), which describe the ‘within-sample’ heterogeneity of ecological communities, as outcome. Outliers +/− five standard deviations were removed from each alpha diversity metric before analysis. CPET parameters were log-transformed, normalized and cleaned from outliers (+/− five standard deviations) for all analyses. All regression analyses included the following possible confounding factors: age, sex, BMI, smoking status, glycated haemoglobin (HbA1c) levels, metformin usage, aspirin usage, PPI usage, diet (FFS), cohort and sequencing batch^2,6,25^. All p-values derived from taxon-trait associations were adjusted for multiple testing using the method of Benjamini and Hochberg to avoid alpha error inflation and thereafter called q-values. P- or q-values < 0.05 were considered significant.

## Results

Characteristics of all 3,558 study participants stratified by quartiles of VO_2peak_ values are provided in Table 1 and post-hoc analyses in Supplementary Table 1. The median age decreased from the first to the fourth quartile of VO_2peak_. There were more women in the first quartile than in the other quartiles. Individuals in the fourth quartile of VO_2peak_ had a higher BMI and lower quality of diet (FFS). Moreover, they also had lower Hba1c levels and use of Metformin. VO_2peak_ was strongly positively correlated with other CPET markers such as VO_2_@AT (*r* = 0.80, *p* < 0.001), Watt_max_ (*r* = 0.92, *p* < 0.001), and peak O_2_ pulse (*r* = 0.82, *p* < 0.001) but inversely with VE/VCO_2_ slope (*r* = − 0.30, *p* < 0.001).

**Table 1:** Characteristics of the study sample stratified by quartiles of peak oxygen uptake (VO_2peak_) values (*n* = 3,558).

Parameter	First quartile	Second quartile	Third quartile	Fourth quartile	Total	*p*-value*
N (%)	890	889	889	890	3,558	–
VO_2peak_ (L/min)	0.58 to 1.48	1.49 to 1.84	1.85 to 2.34	2.35 to 4.68	0.80 to 4.68	–
Age (years)	63 (53–70)	56 (45–65)	54 (43–64)	46 (38–55)	55 (44–65)	**< 0.001**
Female sex (%)	84.8	71.5	38.1	5.2	49.9	**< 0.001**
Body mass index (kg/m^2^)	26.7 (23.6–30.2)	27.3 (24.3–30.6)	27.8 (25.1–30.9)	27.8 (25.4–30.8)	27.4 (24.7–30.6)	**< 0.001**
HbA1c (%)	5.3 (5.0–5.8)	5.3 (4.9–5.7)	5.3 (4.9–5.7)	5.2 (4.9–5.6)	5.3 (4.9–5.7)	**< 0.001**
Metformin usage (%)	6.3	4.7	4.3	2.8	4.5	**0.005**
Smoking (%)	16.9	22.5	22.4	20.9	20.7	**0.010**
Diet (food frequency score)	15.0 (13.0–17.0)	15.0 (13.0–17.0)	14.0 (12.0–16.0)	13.0 (11.0–15.0)	14.0 (12.0–17.0)	**< 0.001**

VO_2peak_ is given as the range within each quartile (min–max). Age, body mass index, glycated haemoglobin (HbA1c, %), food frequency score (FFS), and VO_2peak_ are given as median (1st−3rd quartile). Female sex, metformin usage, and smoking are stated as percentages. *p-values are based on the chi-squared test for categorical variables and the Kruskal–Wallis test for continuous variables. Post-hoc analyses can be found in Supplemental Table 1. *N* Number of cases. Significant values are in bold.

### Associations of CPET with gut microbiota beta diversity

**Fig. 1:**
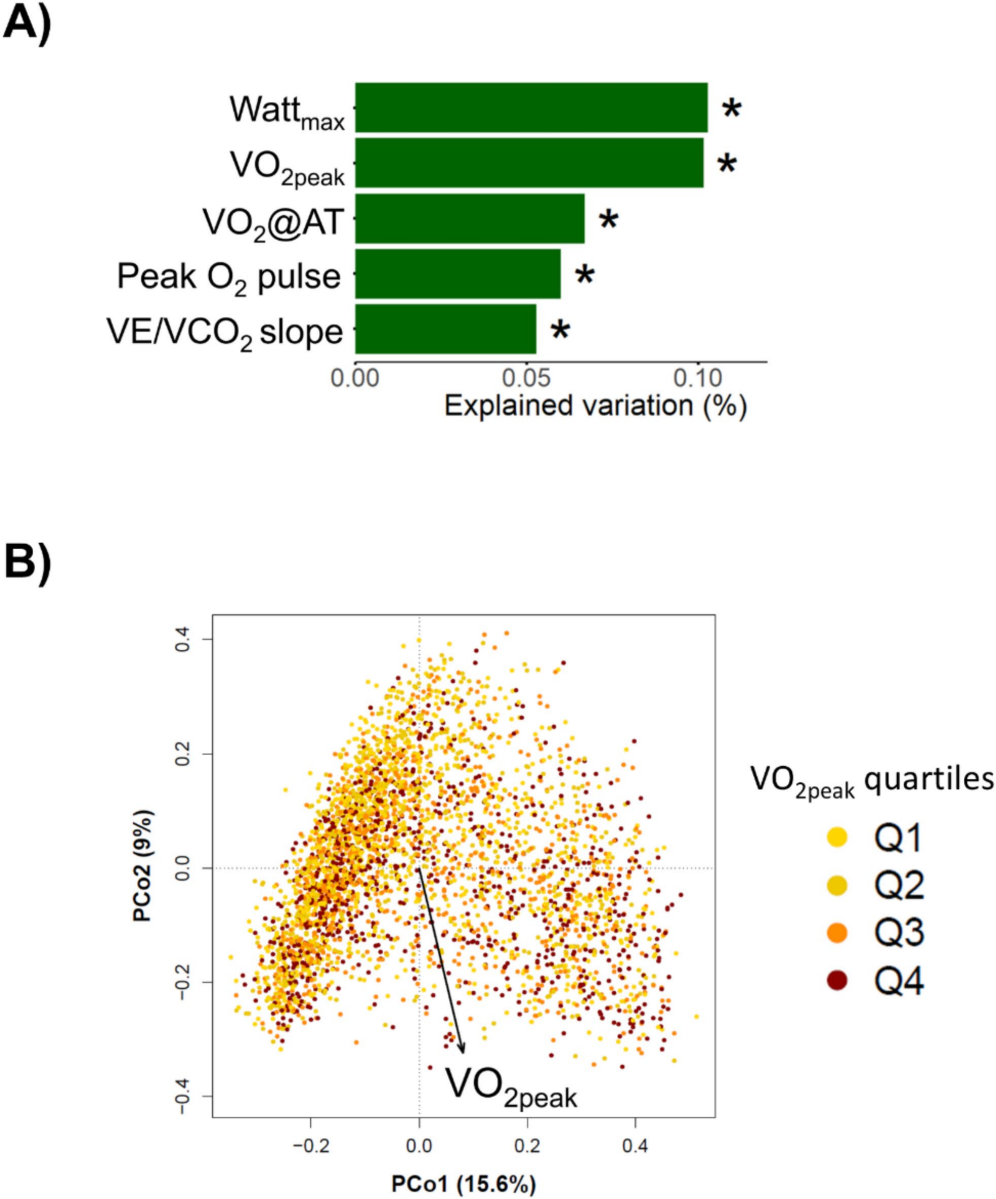
(**A**) Associations between maximum working capacity (Watt_max_), peak oxygen uptake (VO_2peak_), oxygen uptake at the anaerobic threshold (VO_2_@AT), peak oxygen pulse (peak O_2_ pulse) and ventilatory efficiency (VE/VCO_2_ slope) and the gut microbiome. * indicates significant results (*p* < 0.05). Statistical significance was assessed performing permutational analysis of variance. (**B**) Principal coordinate analysis (PCoA) of 3,558 gut microbiota samples based on a Bray-Curtis dissimilarity matrix. The samples are colour coded according to their VO_2peak_ quartile. The arrow denotes the direction of the association between VO_2peak_ and the gut microbiome.

The five most abundant taxa in the complete dataset were *Bacteroides*, *Prevotella*, *Faecalibacterium*, unclassified *Ruminococcacae* and unclassified *Lachnospiraceae*. A total of 97 taxa were present in at least 10% of all samples of which 79 taxa were classified at genus and nine at family level. Further details about the microbiota composition in the analyzed cohorts and the distributions of the individual taxa are given in Supplementary Table 2. Permutational analysis of variance based on a Bray-Curtis dissimilarity revealed significant associations of Watt_max_ (*p* < 0.001), VO_2peak_ (*p* = 0.002), VO_2_@AT (*p* = 0.018), peak O_2_ pulse (*p* = 0.024), and VE/VCO_2_ slope (*p* = 0.034) with the overall microbiome community structure (Fig. 1). Using weighted UniFrac distance as marker of beta diversity yielded significant associations between the gut microbiome and Watt_max_ (*p* = 0.031) and VO_2peak_ (*p* = 0.017) (Fig. S1).

### Taxon associations between CPET and gut microbiota

**Fig. 2:**
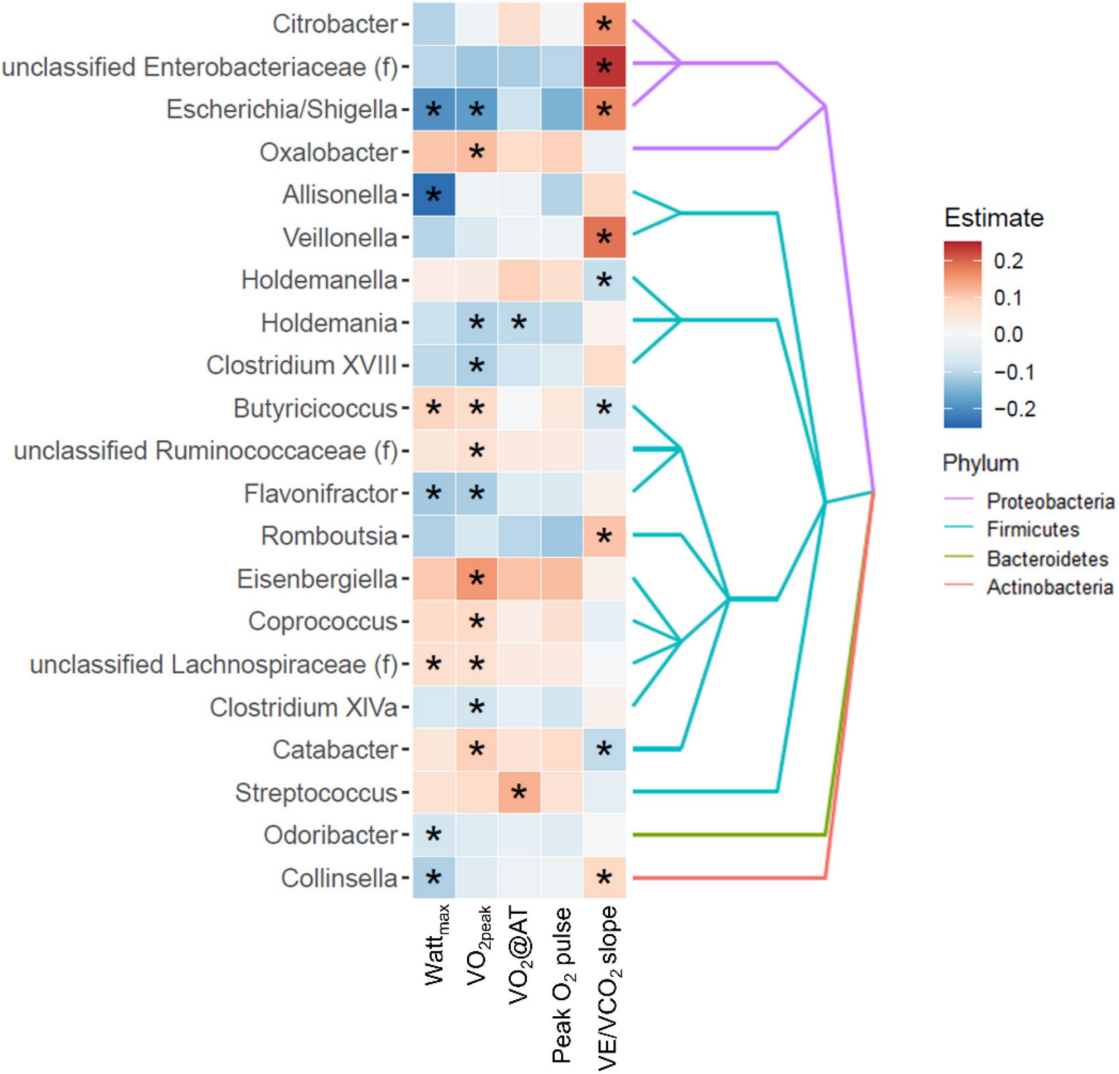
Associations between maximum working capacity (Watt_max_), peak oxygen uptake (VO_2peak_), oxygen uptake at the anaerobic threshold (VO_2_@AT), peak oxygen pulse (peak O_2_ pulse) and ventilatory efficiency (VE/VCO_2_ slope) and individual taxa. The heatmap displays the positive (red) or inverse (blue) associations of the different cardiopulmonary exercise testing (CPET) parameters Watt_max_, VO_2peak_, VO_2_@AT, peak O_2_ pulse and VE/VCO_2_ slope with individual genera or families. Only taxa with at least one significant association result to any of the CPET variables are shown. *Indicates significant results (q < 0.05). (f): Family. Statistical significance was assessed using a negative binomial regression model.

We found, in negative-binomial regression models, that VO_2peak_ and Watt_max_ had numerous associations with the gut microbiome (Fig. 2 and Supplementary Table 3). Both variables showed inverse associations with *Flavonifractor* and the opportunistic Gram-negative pathogen *Escherichia/Shigella* as well as positive associations with several short-chain fatty acid (SCFA) producers such as *Butyricoccus*, unclassified *Ruminococcaceae*, *Coprococcus*, and unclassified *Lachnospiraceae*. Additionally, VO_2_@AT showed an inverse association with *Holdemania* whereas peak O_2_ pulse did not show any significant association. Of note, VE/VCO_2_ slope exhibited opposite associations with the gut microbiota when compared to VO_2peak_ and Watt_max_ including strong positive associations with *Veillonella* and the opportunistic pathogens *Citrobacter*, unclassified *Enterobacteriaceae*, and *Escherichia/Shigella*.

**Fig. 3:**
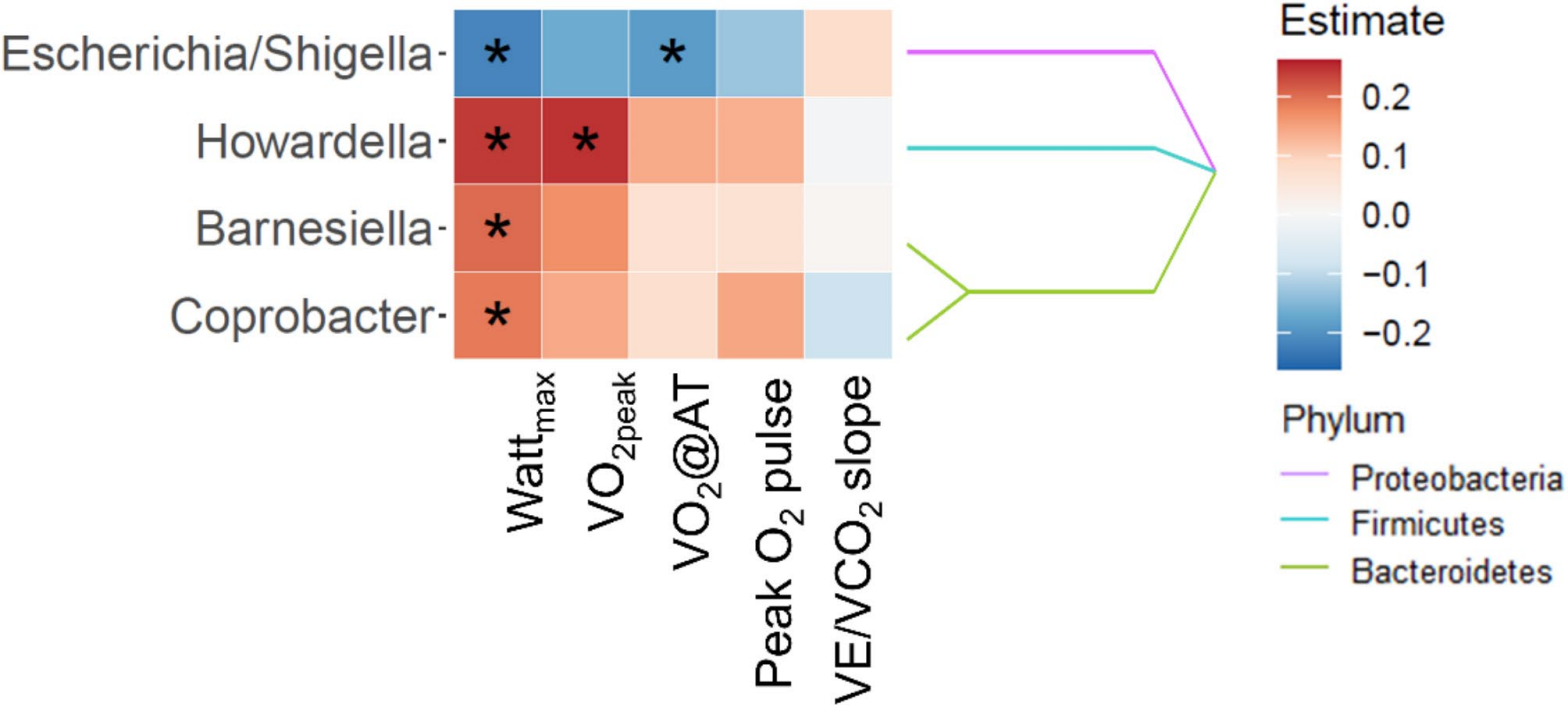
Associations between maximum working capacity (Watt_max_), peak oxygen uptake (VO_2peak_), oxygen uptake at the anaerobic threshold (VO_2_@AT), peak oxygen pulse (peak O_2_ pulse) and ventilatory efficiency (VE/VCO_2_ slope) and microbial presence-absence patterns. The heatmap displays the positive (red) or inverse (blue) associations of the different cardiopulmonary exercise testing (CPET) parameters Watt_max_, VO_2peak_, VO_2_@AT, peak O_2_ pulse and VE/VCO_2_ slope with individual genera or families based on presence-absence microbial abundance data. Only taxa with at least one significant association result to any of the CPET variables are shown. * Indicates significant results (q < 0.05). Statistical significance was assessed using a logistic regression model.

A second model, using logistic regression models, analysed the associations of CPET with the presence-absence data of the gut microbiota (Fig. 3 and Supplementary Table 4). Watt_max_ and VO_2_@AT showed an inverse association with the presence of the opportunistic Gram-negative pathogens *Escherichia/Shigella*. Watt_max_ showed additional positive associations with the presence of *Howardella*, *Barnesiella*, and *Coprobacter*. VO_2peak_ was also positively associated with Howardella. Peak O_2_ pulse and VE/VCO_2_ slope did not show any significant associations with microbial taxa in this model.

### Associations of CPET with microbial alpha diversity

**Fig. 4:**
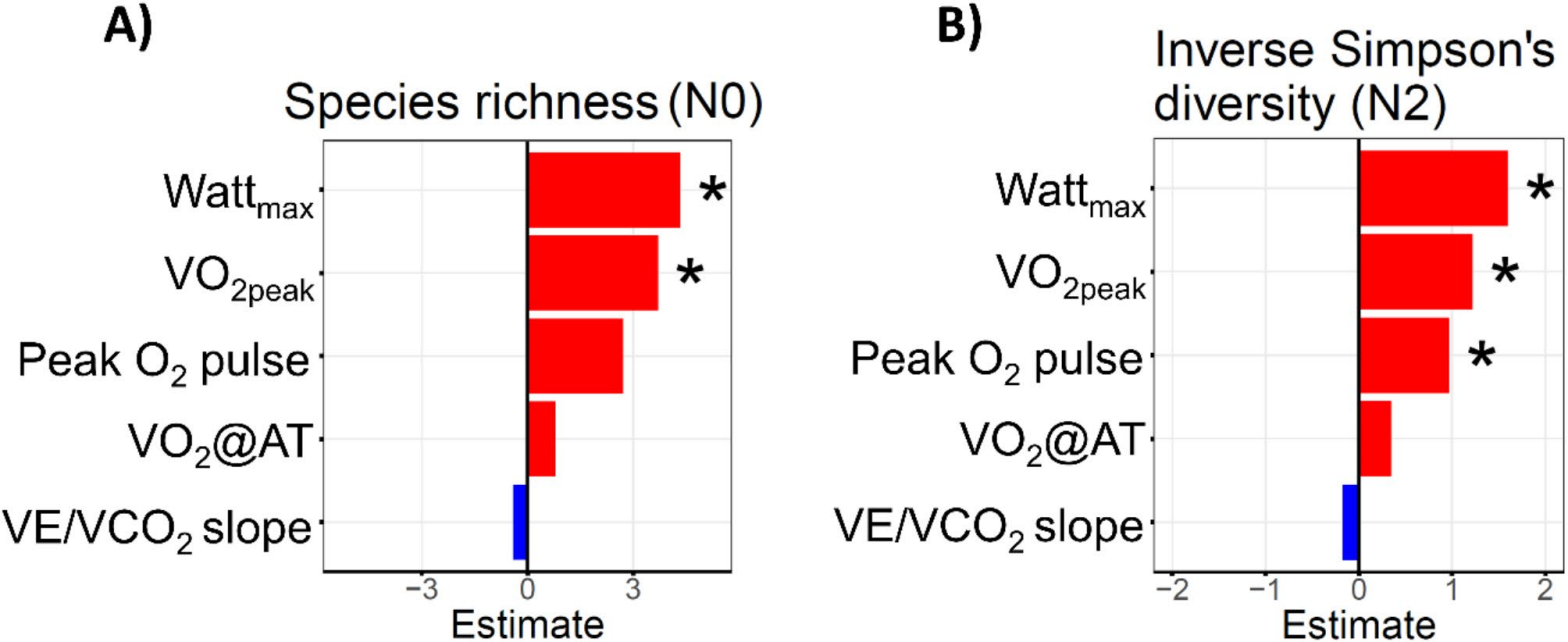
Associations between maximum working capacity (Watt_max_), peak oxygen uptake (VO_2peak_), oxygen uptake at the anaerobic threshold (VO_2_@AT), peak oxygen pulse (peak O_2_ pulse) and ventilatory efficiency (VE/VCO_2_ slope) and microbial alpha diversity. The barplot displays positive (red) or inverse (blue) associations of the different cardiopulmonary exercise testing (CPET) parameters Watt_max_, VO_2peak_, VO_2_@AT, peak O_2_ pulse and VE/VCO_2_ slope with alpha diversity scores species richness (N0) and inverse Simpson’s index (N2). * Indicates significant results (*p* < 0.05). Statistical significance was assessed using a linear regression model.

The distributions of species richness and inverse Simpson’s index in the complete dataset were 245.5 (203.0–294.0, IQR) and 42.0 (30.4–57.2, IQR), respectively. Watt_max_ (*p* = 0.013) and VO_2peak_ (*p* = 0.034) showed positive associations with species richness (Fig. 4A). Moreover, Watt_max_ (*p* = 0.002), VO_2peak_ (*p* = 0.020), and peak O_2_ pulse (*p* = 0.048) were also associated with the inverse Simpson’s index (Fig. 4B). There were no significant associations of VO_2_@AT and VE/VCO_2_ slope with alpha diversity.

## Discussion

Our gut is colonized by numerous microorganisms that exert important metabolic functions. Changes in the composition of these microbes can either have beneficial or detrimental effects on our health. Interestingly, the gut microbiome can be affected by diet and exercise and through its metabolites may influence the cardiovascular risk^26^. A healthy gut microbiome is characterized by potentially beneficial microorganisms that act as a deterrent to possible pathogenic strains, competing for resources and avoiding invasion of foreign microbes^27^.

Our findings suggest that low CRF, which is common in the general population due to sedentary lifestyle of most individuals throughout life, seems to be associated with a disadvantageous gut microbiome constituted by potential harmful microorganisms. Specifically, lower CRF (assessed by standardized CPET) was associated with an altered gut microbiome characterized by reduced alpha diversity that included a declined abundance of SCFA-producers^28,29^ such as *Butyricoccus*, unclassified *Ruminococcaceae*, *Coprococcus*, or unclassified *Lachnospiraceae*, and an increased abundance of *Holdemania* and *Veillonella* as well as several opportunistic Gram-negative pathogens like *Escherichia/Shigella*, *Citrobacter*, and unclassified *Enterobacteriaceae*. This microbiota pattern may be associated with a potentially proinflammatory environment that putatively contributes to the development of cardio-metabolic diseases such as obesity, type 2 diabetes mellitus, non-alcoholic fatty liver disease and atherosclerosis^30^.

While higher microbial diversity leads to competition between beneficial and potentially pathological species inhibiting the growth of the latter, a reduced diversity or overgrowth by pathological species can promote a “leaky gut” where adverse metabolites, secreted by the bacteria, leave the gut into the systemic circulation^27^. Particularly, gastrointestinal or metabolic disorders are associated with low gut microbiota diversity^2,23,31^ which can be followed by microbiome instability, a phenotype characterized by a further increase in opportunistic pathogens and the corresponding potential for secretion of proinflammatory lipopolysaccharides (LPS) over time^6^. Consequently, the presence of reduced microbial diversity in individuals with low CRF may trigger systemic inflammation and accelerate atherosclerosis^32^. Besides that, our digestive system does not have the capacity to break down some components of dietary fibres because of the lack of specific enzymes. Contrary to that, some microorganisms have the ability to ferment these fibres, including otherwise indigestible carbohydrates, into absorbable forms of SCFA (mainly acetate, propionate, and butyrate). SCFA decrease the pH in the gut lumen, which inhibits the growth of pathogens^33^. Moreover, SCFA do also have anti-inflammatory and immunomodulatory effects^27,34^.

We have also found that lower CRF was associated with a greater abundance or presence of several opportunistic Gram-negative pathogens like *Escherichia/Shigella*, *Citrobacter*, and other unclassified *Enterobacteriaceae*. The endotoxin LPS is a central component of the Gram-negative bacterial cell wall. While under physiological conditions a healthy gut barrier prevents against the transit of LPS into circulation, the presence of pathogenic bacteria may disrupt this barrier and facilitate its transit^27,35^. Moreover, LPS can bind toll-like receptor 4 (TLR-4) which is expressed on immune cells promoting local and systemic inflammation^27,36^. Besides that, *Escherichia/Shigella* and *Citrobacter* have the capacity to metabolize dietary choline, carnitine and betaine, that are present in red meat and fish, into trimethylamine which is then converted into trimethylamine *N*-oxide (TMAO) by flavin monooxygenases present in the liver^37,38^. The presence of high serum TMAO levels is considered to be associated with endothelial dysfunction, vascular inflammation and atherosclerosis^39–42^. Finally, it is important to consider that while a gut microbiome enriched with proinflammatory opportunistic pathogens results in subclinical systemic inflammation that may lead to cardiometabolic diseases, it may be also deleteriously shaped by the inflammation, causing a vicious cycle.

In a much-noticed recent study, Scheimann et al. have found higher levels of *Veillonella atypica* in the gut microbiome of marathon runners^43^. Transferring this strain into mice was associated with an increase in physical performance. The authors further found that lactate translocated into the gut lumen after exercise which could then be metabolized by *Veillonella* and used as an energy source. They concluded that *Veillonella atypica* may enhance the athletic performance. However, in contrast to that study, we found higher levels of *Veillonella* in individuals with lower CRF. Microbial mechanisms that enhance the physical performance in regularly exercising athletes may not work similarly in sedentary individuals, as sedentarism, and its subsequent pathophysiological mechanisms, is not necessarily the opposite of those related with physical activity or exercise training^10^.

### Potential mechanisms for the observed associations

The precise mechanisms explaining the associations of lower CRF with an altered gut microbiota characterized by an increase in opportunistic pathogens, a decline in SCFA-producers and low microbial alpha diversity are difficult to clarify given the cross-sectional nature of our study. Lower CRF and the aforementioned detrimental gut microbiome changes share multiple risk factors and comorbidities, such as older age, obesity, smoking, impaired glucose tolerance and diet that may explain these associations as parallel changes instead of causal ones. On the other hand, we have incorporated these risk factors as possible confounders in our regression models without a significant modification of our results. This suggests a direct association of lower CRF with and the gut microbiota independent from the risk factors mentioned above.

Hypothetically, an altered (proinflammatory) gut microbiome could lead to a lower CRF or vice versa. While we cannot surely exclude the first pathway of interacting process direction, our findings seem to support the latter, i.e. that the sedentary lifestyle may result in a detrimental microbiome composition. After a meal, bile acids are excreted into the duodenum to support the emulsification, solubilization and absorption of dietary lipids, fat-soluble vitamins, and cholesterol^30,44^. Bile acids are synthesized by liver cells and secreted with the bile into the small intestine^44,45^. Importantly, besides their role in digestion, bile acids also have toxic and detergents properties with a bactericidal effect decreasing the overall quantity of gut bacteria, thus, probably improving microbial diversity^44,46–48^. A sedentary lifestyle may lead to a decrease in high-density lipoprotein-cholesterol levels increasing the cholesterol saturation of the bile^49,50^. Besides that, sedentarism is usually associated with insulin resistance^50,51^ that leads to a hyperinsulinemic state which promotes an uptake of cholesterol by liver cells^50,52^ resulting in increased cholesterol levels in the biliary tract and lower secretion of biliary acids^50,53,54^. In addition, there is also a decrease in gallbladder motility through lower cholecystokinin secretion^50,55^. Taken together, these processes may change the detergent bacterial characteristics of the bile acids. Consequently, a lower CRF may be associated with deleterious changes in bile acid secretion and together with an increased gastrointestinal transit time^56^ this may result in an altered gut microbiome—“the sedentary’s gut microbiome”.

### Study limitations

There are some limitations of our study that need to be mentioned. First, due to the cross-sectional design, causal inferences cannot be made and further replication studies are warranted to better understand the mechanisms behind these associations. Second, our study sample comprised only Caucasians individuals of European ancestry; therefore, extrapolation to other ethnicities, different dietary habits or age groups is not possible. Third, even though we incorporated several confounders in our multivariable regression models, we cannot exclude unmeasured or unknown residual confounding. Finally, in this study an EDTA buffer was used to preserve the microbial composition of the faecal samples which may affect the abundance of specific bacterial groups depending on the time interval between sample collection and processing^57^. In spite of these limitations, our analyses have also significant strengths, including the large number of individuals from the general population with a wide age range, the standardized assessment of CRF by CPET and the gut microbiome as well as the availability of data on multiple metabolic risk factors that could be adjusted for.

## Conclusions

Our findings from a large population-based sample demonstrated that lower CRF was associated with a detrimental gut microbiome composition characterized by reduced microbial diversity, loss of beneficial and an increase in potentially pathogenic bacteria that may have further deleterious effects for the human health—“the sedentary’s gut microbiome”.

## Electronic supplementary material

Below is the link to the electronic supplementary material.

Supplementary Material 1

Supplementary Material 2

## Data Availability

The datasets generated during and/or analyzed during the current study are not publicly available due to data protection aspects but are available in an anonymized form from the corresponding author on reasonable request.

## Abbreviations

BMI: Body mass index
CPET: Cardiopulmonary exercise testing
CRF: Cardiorespiratory fitness
FFS: Food frequency score
HbA1c: Glycated haemoglobin
LPS: Lipopolysaccharides
Peak O_2_ pulse: Peak oxygen pulse
RDP: Ribosomal Database Project
SCFA: Short-chain fatty acid
SHIP: Study of Health in Pomerania
TLR-4: Toll-like receptor 4
TMAO: Trimethylamine N-oxide
VCO_2_: Carbon dioxide output
V_E_: Minute ventilation
VO_2_: Oxygen uptake
VO_2peak_: Peak oxygen uptake
VO_2_@AT: Oxygen uptake at the anaerobic threshold
V-slope method: Relation of VO_2_ to VCO_2_
VE/VCO_2_ slope: Ventilatory efficiency
Watt_max_: Maximum working capacity
