# Supplementary material for: Lower cardiorespiratory fitness is associated with an altered gut microbiome. The Study of Health in Pomerania (SHIP)
Source: Sci Rep. 2025 Feb 12;15:5171. doi: 10.1038/s41598-025-88415-4 (PMC11822121; doi:10.1038/s41598-025-88415-4)
Supplement: Supplementary file 2 — Supplementary Material 2 [file 41598_2025_88415_MOESM2_ESM.pdf]

Supplementary Figures

Lower cardiorespiratory fitness is associated with an altered gut microbiome – “The Sedentary’s Gut Microbiome”

Marcello Ricardo Paulista Markus[1, 2, 3*]; Frank-Ulrich Weiss[4]; Johannes Hertel[5]; Stefan Weiss[4]; Malte Rühlemann[6,7]; Corinna Bang[6]; Andre Franke[6]; Uwe Völker[8]; Georg Homuth[8]; Thomas Kocher[9]; Henry Völzke[2, 10]; Markus M. Lerch[4]; Till Ittermann[2, 10]; Stephan Burkhard Felix[1, 2]; Ralf Ewert[1]; Martin Bahls[1, 2]; Marcus Dörr[1,2]; Fabian Frost[4]

[1] Department of Internal Medicine B, University Medicine Greifswald, Greifswald, Germany.

[2] German Centre for Cardiovascular Research (DZHK), partner site Greifswald, Greifswald, Germany.

[3] German Center for Diabetes Research (DZD), partner site Greifswald, Greifswald, Germany.

[4] Department of Medicine A, University Medicine Greifswald, Greifswald, Germany.

[5] Department of Psychiatry and Psychotherapy, University Medicine Greifswald, Greifswald, Germany.

[6] Institute of Clinical Molecular Biology, Christian-Albrechts University of Kiel, Kiel, Germany.

[7] Institute for Medical Microbiology and Hospital Epidemiology, Hannover Medical School, Hannover, Germany

[8] Department of Functional Genomics, Interfaculty Institute of Genetics and Functional Genomics, University Medicine Greifswald, Greifswald, Germany.

[9] Unit of Periodontology, Department of Restorative Dentistry, Periodontology, Endodontology, and Preventive and Pediatric Dentistry, University Medicine Greifswald, Greifswald, Germany.

[10] Department of Study of Health in Pomerania/Clinical-Epidemiological Research, Institute for Community Medicine, University Medicine Greifswald, Greifswald, Germany.

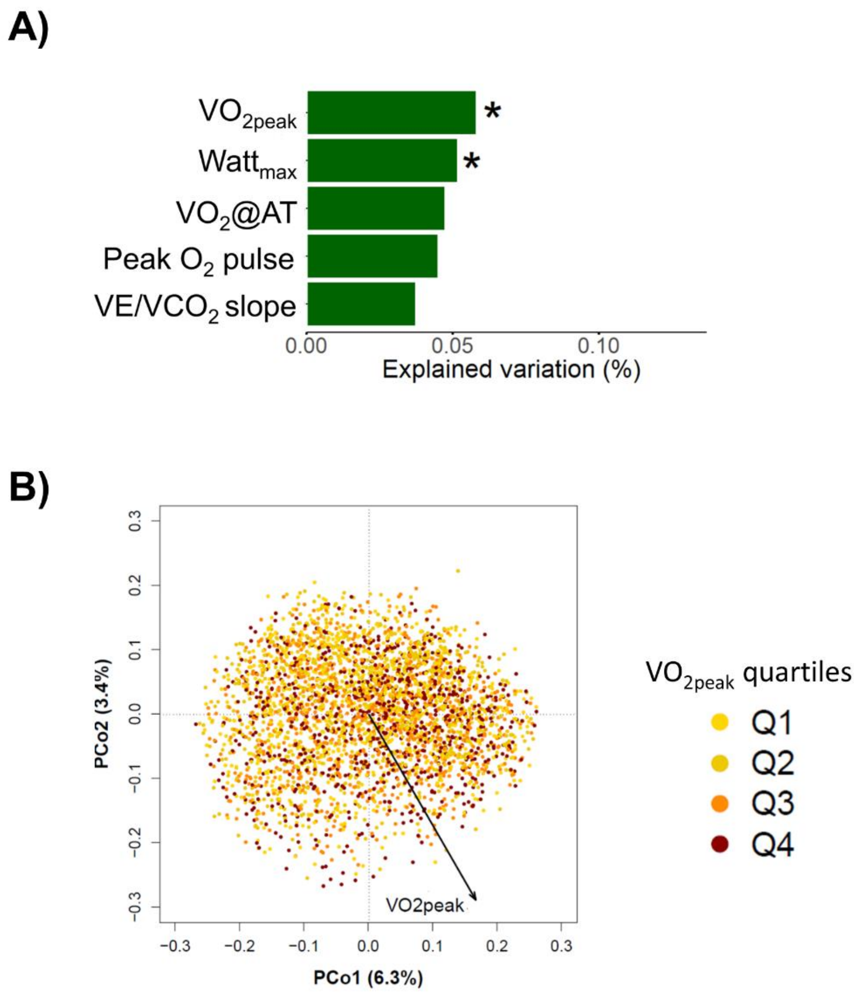

Figure S1: (A) Associations between maximum working capacity ($Watt_{max}$), peak oxygen uptake (VO_{2peak}), oxygen uptake at the anaerobic threshold (VO_2@AT), peak oxygen pulse (peak O_2 pulse) and ventilatory efficiency (VE/VCO_2 slope) and the gut microbiome. * Indicates significant results ($p<0.05$). Statistical significance was assessed performing permutational analysis of variance. **(B)** Principal coordinate analysis (PCoA) of 3,616 gut microbiota samples based on a weighted UniFrac distance matrix. The samples are colour coded according to their VO_{2peak} quartile. The arrow denotes the direction of the association between VO_{2peak} and the gut microbiome.
